# Longitudinal mental health data collected via the Corona Health smartphone app during COVID-19

**DOI:** 10.1038/s41597-026-07015-7

**Published:** 2026-03-11

**Authors:** Michael Winter, Carsten Vogel, Johannes Schobel, Miriam Schlüter, Harald Baumeister, Yannik Terhorst, Winfried Schlee, Berthold Langguth, Peter Heuschmann, Caroline Cohrdes, Rüdiger Pryss

**Affiliations:** 1 Institute for Clinical Epidemiology and Biometry, University of Würzburg, Würzburg, Germany; 2 Institute of Medical Data Science, University Hospital of Würzburg, Würzburg, Germany; 3 Institute DigiHealth, Neu-Ulm University of Applied Sciences, Neu-Ulm, Germany; 4 Department of Clinical Psychology and Psychotherapy, Ulm University, Ulm, Germany; 5 German Center for Mental Health (DZPG), Partner-Site Mannheim-Heidelberg-Ulm, Ulm, Germany; 6 German Center for Child and Adolescent Health (DZKJ), Partner-Site Ulm, Ulm, Germany; 7 Department Psychologie, LMU München, Munich, Germany; 8 Deutsches Zentrum für Psychische Gesundheit (DZPG), Standort München-Augsburg, Augsburg, Germany; 9 Institute for Information and Process Management, Eastern Switzerland University of Applied Sciences, St. Gallen, Switzerland; 10 Department of Psychiatry and Psychotherapy, University of Regensburg, Regensburg, Germany; 11 Department 2 Epidemiology and Health Monitoring, Robert Koch Institute, Berlin, Germany

## Abstract

Mental health impacts during the COVID-19 pandemic underscored the importance of real-time assessment methods to capture population-level changes (e.g., longitudinal changes in quality of life). This dataset contains questionnaire responses collected with the Corona Health app, a multilingual mHealth app available on Android and iOS platforms. The dataset includes baseline from 2,704 participants (i.e., adults aged 18 years and older, living in Germany) and 11,541 repeated ecological momentary assessment (EMA) responses, providing longitudinal mental health data throughout various phases during the pandemic period (i.e., data collected between July, 2020 and January, 2025). The questionnaires assessed domains such as quality of life, psychological well-being, coping mechanisms, and pandemic-related concerns. In addition to questionnaire responses, the dataset includes sensor data such as GPS location information and app usage statistics collected with participant consent. The described dataset enables researchers to examine mental health trajectories during and after COVID-19, analyze relationships between psychological factors and pandemic experiences, and investigate patterns in longitudinal mental health data.

## Background & Summary

The COVID-19 pandemic represented a global health crisis that altered daily life worldwide^1^. Beyond the direct health impacts of the coronavirus, the pandemic triggered widespread implementation of public health measures including lockdowns, social distancing, quarantine protocols, and restrictions on social gatherings^2^. These necessary containment strategies, while crucial for controlling viral transmission, created secondary consequences for population mental health^3–5^. The sudden disruption of social connections, economic uncertainty, and prolonged periods of isolation contributed to temporary increases in symptoms of anxiety, depression, and psychological distress across diverse populations, that have not not returned to pre-pandemic levels yet^6^. Moreover, traditional face-to-face mental health services became limited or inaccessible.

In response to this challenge, the Corona Health mHealth app was developed to capture various aspects of individuals’ pandemic experiences and mental health outcomes^7^. Through EMAs, the mHealth app enabled real-time data collection by prompting users to report their experiences and emotions as they occurred in their natural environments. By utilizing smartphone technology and a momentary assessment approach, Corona Health aimed to bridge the gap between mental health monitoring needs and limited traditional clinical resources during the pandemic. Moreover, a crucial function of Corona Health was that participants received immediate automated feedback on their psychological well-being based on their responses. For example, participants were advised to seek help and where to get more information about support options, such as a crisis hotline, if they exceeded a clinically significant cut-off.

The Corona Health platform hosted multiple concurrent studies addressing different aspects of pandemic-related health and well-being. This dataset specifically contains data from the “Mental Health for Adults (18 Years +)” study, one of five studies implemented within Corona Health^8^. The dataset comprises from 2,704 participants baseline as well as 11,541 repeated EMA responses collected over nearly five years (July 2020 to January 2025), spanning multiple pandemic phases from initial outbreak through longer-term adaptation periods. With multilingual support and both self-reported questionnaire data and objective sensor measurements, the dataset provides statistical power for examining mental health responses and behavioral patterns across diverse population groups and pandemic phases.

Moreover, this dataset offers potential for secondary analysis across multiple research domains. Mental health researchers can utilize the longitudinal data to investigate psychological resilience patterns and examine temporal dynamics of psychopathological symptoms and well-being indicators during prolonged crisis periods. The data enables deeper insights into the distribution of mental health problems, needs, and at-risk groups, as well as associations between a comprehensive set of mental health indicators over time, including analyses in conjunction with behavioral smartphone sensing data. Digital health researchers can evaluate smartphone-based mental health assessment approaches and examine user engagement patterns with health apps during crisis periods, while EMA methodology researchers can analyze compliance patterns and optimal assessment frequencies in longitudinal smartphone-based studies.

The comprehensive, multilingual dataset thus provides a valuable resource for advancing the understanding of pandemic mental health impacts and digital health monitoring approaches. It contributes to a growing body of openly available, high-quality psychological and behavioral data collected during the COVID-19 pandemic. Related efforts include the COVID-19 Snapshot Monitoring (COSMO) dataset from Germany, which offers repeated cross-sectional insights into public risk perceptions, behaviors, and attitudes over 69 survey waves^9^. Further data comprises information about the impact of the COVID-19 pandemic from individuals in France^10^. Moreover, a dataset from Turkey focusing on distress intolerance and anxiety across pandemic^11^ and a longitudinal observation on the change in psychosocial factors in Japan during the pandemic are other related contributions^12^.

The present dataset complements these efforts through several distinctive features: it combines validated clinical instruments (e.g., PHQ-9, GAD-7) with passive smartphone sensing data (i.e., GPS location and app usage statistics), enabling joint analysis of self-reported mental health outcomes and objective behavioral indicators. Furthermore, unlike cross-sectional designs, this dataset captures within-person longitudinal trajectories via repeated EMA assessments over nearly five years, providing granular insight into individual-level mental health dynamics across multiple pandemic phases. This integration of EMA methodology with mobile sensing in a single longitudinal framework offers unique opportunities for research at the intersection of digital phenotyping and mental health monitoring. Together, these initiatives enhance international comparative research and provide context for studying the psychological, behavioral, and digital health dimensions of crisis response.

### Previous Publications Using This Dataset

The comprehensive nature and extended timeframe of the Corona Health app have made it a valuable resource for the scientific community, resulting in multiple publications that have contributed to our understanding of pandemic-related mental health outcomes. A total of 17 publications have utilized data from the broader Corona Health app^7,13–19^, with 9 publications specifically drawing from the “Mental health for adults (18 years +)” dataset presented in this paper. These papers used the dataset at different points in time (e.g., while the app was still active), and therefore the data considered may differ based on several factors (e.g., only specific items were used, or only a particular population was analyzed), which are briefly described in the following and summarized in Table 1.

**Table 1:** Summary of Studies on Smartphone Use, Mental Health, and Quality of Life Based on Data from the “Mental Health for Adults (18 Years +)” study.

Study	Sample Size	Focus	Method	Key Findings
Cohrdes *et al*. (2024)	2,137	Quality of life trajectories during COVID-19	Latent class analysis	Identified four patterns (resilient, recovering, delayed, chronic); delayed group most affected.
Simon *et al*. (2024)	752	Smartphone usage and insomnia	Machine learning on smartphone features	Weak correlations; limited predictive value for insomnia detection using usage data alone.
Edler *et al*. (2024a)	249	GPS-based regional factors and depression	Machine learning with regional data	COVID-19 rates, nursing home capacity, and parental benefit ratios predicted depressive symptoms.
Edler *et al*. (2024b)	490	Smartphone social interaction and mental health	Regression analysis	Messenger use negatively predicted depression and anxiety; video calls linked to depression; SMS to anxiety.
Cohrdes *et al*. (2023)	2,137	Coping strategies and quality of life	Cross-sectional analysis	Support- and meaning-focused coping improved QoL; escape-avoidance reduced it; effects moderated by age, sex, education.
Weiss *et al*. (2022)	486	Extraversion and social media effects on mental health	Moderation analysis	Extraversion buffered general mental health, but amplified social media’s negative effect on depression.
Mulansky *et al*. (2022)	627	Social media use and depression	Correlational analysis	Higher weekly usage time associated with increased depression symptoms.
Wetzel *et al*. (2021)	364	Smartphone communication, loneliness, and well-being	Lifespan analysis	Younger adults: more use linked to more loneliness; older adults showed opposite pattern.
Eicher *et al*. (2021)	1,396	Quality of life and pandemic conditions	WHOQOL	Women, younger adults, and those with work disruptions had lower QoL; outdoor access and hybrid work improved outcomes.

Research has explored smartphone data and mental health outcomes with mixed results. One study of 752 participants found limited predictive value of smartphone usage features for detecting insomnia symptoms^20^. Investigation of GPS-based environmental factors in 249 participants revealed that regional characteristics like COVID-19 infection rates could predict depressive symptoms^21^. Analysis of smartphone social interaction data from 490 participants showed messenger use negatively predicted both depressive and anxiety symptoms^22^.

Studies on social media revealed concerning patterns: extraversion paradoxically amplified negative effects of social media use on depression in 486 participants^23^, while research with 627 participants confirmed a positive association between social media usage and depression symptoms^24^. Smartphone communication patterns in 364 participants showed age-dependent relationships with loneliness and social well-being^25^.

Regarding quality of life during the COVID-19 pandemic, latent class analysis of 2,137 German adults identified four distinct patterns (resilient, recovering, delayed, chronic), with the delayed class showing steepest decline and slowest recovery^26^. Further analysis of this cohort found support- and meaning-focused coping beneficial for quality of life, while escape-avoidance coping showed strong negative associations^27^. A comprehensive assessment of 1,396 participants revealed diminished quality of life among women, younger individuals, and those with pandemic-related employment disruptions^28^.

This release provides the complete, longitudinal dataset spanning the full collection period (July 2020 to January 2025), including all baseline assessments (n = 2,704), all repeated EMA responses (n = 11,541), and associated sensor data (i.e., GPS and app usage statistics). Unlike the prior publications, which analyzed temporal snapshots or specific variable subsets, this release enables: (1) comprehensive replication and extension of previous findings; (2) novel analyses across the full pandemic timeline, including understudied recovery and adaptation phases; (3) integration of questionnaire and sensor data that were not jointly analyzed in prior work; and (4) methodological investigations of EMA compliance and engagement patterns across the complete study duration.

## Methods

### Study Design and App Framework

All the data was collected using the Corona Health mHealth app. The app was publicly available on Android and iOS platforms, and supported multilingual deployment across eight languages (i.e., German, English, Spanish, French, Hungarian, Italian, Russian, Serbia). The app enabled real-time data collection of self-reported mental health measures and sensor-based contextual data. In general, the technical platform of Corona Health followed a modular design, hosting five distinct research studies focusing on various aspects of mental and physical health outcomes in adults and adolescents:

- Mental Health for Adults (18 Years +; subject of this paper)
- Mental Health for Adolescents (12 to 17 Years)^14^
- Physical Health for Adults (18 Years +)^13^
- Recognizing Stress for Adults (18 Years and up)^29^
- Acceptance of Pandemic Apps^30^

Corona Health was developed based on the TrackYourHealth framework^15,31^, which provided robust support for EMA studies, including multilanguage content delivery, sensor integration, and compliance with data privacy and regulatory standards (e.g., General Data Protection Regulation and Medical Device Regulation in the European Union). All user data were stored anonymously, with mobile sensing features activated only upon user consent.

This dataset specifically contains data from the “Mental Health for Adults (18 years +)” study only. Data collection for this specific study spanned nearly five years, from July 21, 2020, to January 25, 2025, covering multiple phases of the COVID-19 pandemic from initial outbreak through longer-term adaptation periods. The dataset comprises 2,704 baseline questionnaire responses and 11,541 EMA responses from 1,488 participants, providing comprehensive longitudinal mental health data throughout the pandemic period. The questionnaires assessed multiple domains including demographics, quality of life, psychological well-being, coping mechanisms, and pandemic-related concerns.

### Input Data

#### Questionnaire Data

Each questionnaire was created via a structured and automated content pipeline. It started with Excel-based templates, selected for their readability and easy editing by non-IT experts. These templates were converted to JSON for automated distribution via a RESTful API. The baseline questionnaire took approximately 20 minutes to complete, while repeated EMAs were approximately 10 minutes. Repeated EMAs were scheduled weekly, based on initially configured schedule or triggered by the users themselves, and they received local push notifications directly from Corona Health accordingly. All responses were collected through the respective smartphone application using a structured JSON format. The questionnaire framework supported multiple question types including multiple choice questions, Likert scale ratings, text input fields, and slider-based responses. The content pipeline system ensured consistency across multiple languages and allowed for dynamic content updates while maintaining questionnaire integrity.

#### Mobile Sensing Data

Mobile sensing was implemented via two mechanisms:

- Opportunistic sensing: GPS-based coarse location data (accuracy limited to 11.1 km) and device operating system metadata were collected automatically during questionnaire completion for participants who provided consent.
- Participatory sensing: Aggregated app usage data (Android only) including screen time and application foreground activity for selected apps (top 5 used apps and predefined social media apps) were collected with explicit user permission.

Sensor data were collected only at the time of questionnaire completion and contingent on user permission. On Android, app usage statistics were obtained through the UsageEvents API provided by the Android operating system, which recorded timestamped application-level foreground and background activity events. These raw events were aggregated on the device into daily-level metrics (e.g., total screen time, per-app usage durations, and activity/inactivity intervals) before transmission to the backend. The specific data collected included:

- Total daily phone usage duration (screen-on time)
- Daily foreground usage time for the five most frequently used apps and predefined social media apps
- Timestamps of first and last usage per tracked application
- Duration of background (foreground service) usage for apps running without visible activity
- Time intervals of user activity and inactivity across the day

For location data, coarse-grained GPS coordinates were obtained via the device’s native location services and were processed to maintain an accuracy of 11.1 km to protect participant privacy while enabling regional analysis. Device information including operating system version and device type were automatically recorded with each questionnaire submission.

### Data Management and Processing

#### Backend Infrastructure and Storage

All data were stored in a relational database with structured schemas for users, questionnaires, and sensor events. The data architecture supported multilingual content and dynamic feedback based on in-app rules. The database used a relational structure with core entities including Users, Studies, Questionnaires, Questionnaire Elements, Answersheets, and Feedback components.

The backend was developed using the Laravel PHP framework and followed RESTful API design principles with JSON:API specification for data exchange^32^. Data collection and study participation were managed through a set of RESTful API endpoints. Participants retrieved available studies via GET /studies/ and accessed study-level metadata through GET /studies/{id}. Enrollment was completed by submitting a subscription request to POST /studies/{id}/subscribe. Questionnaire content, including item structure, question types, response options, and validation parameters, was delivered to the app via GET /questionnaires/{id}/structure. Completed responses were submitted through POST /questionnaires/{id}/answersheets, which triggered the server-side validation pipeline described below.

Further, the database used a relational structure (i.e., MySQL) in which studies were associated with one or more questionnaires, each composed of polymorphic elements (i.e., page breaks, headlines, text fields, and questions). Submitted responses were serialized and stored as JSON objects alongside associated sensor data and client device metadata. The complete entity-relationship model is described in^7^.

This architecture ensured scalable and secure data collection while maintaining system responsiveness across diverse mobile devices and network conditions (more information on requirements and technical implementation are described in^7,31^).

#### Data Anonymization and Quality Assurance

All data were collected and stored anonymously. Participants were assigned anonymous user IDs upon registration, and no personally identifiable information was collected or stored. The anonymous design ensured that individual participants could not be identified from the collected data. However, this approach also had limitations (e.g., it prevented the creation of persistent user profiles). As a result, if a user deleted and reinstalled the Corona Health app or switched to a new smartphone, they were treated as a new user in the dataset. Several measures were implemented to ensure data quality:

- Real-time validation of questionnaire responses within the app
- Automatic detection and handling of incomplete responses
- Timestamp validation for all data entries
- Automated backup and integrity checking procedures
- Offline functionality for the mHealth app to ensure data collection continuity during network interruptions

### Ethics, Regulatory Compliance, and Participant Recruitment

The study was approved by the Ethics Committee of the University of Würzburg (Ref No. 130/20-me). Corona Health complied with the Medical Device Regulation (MDR) and General Data Protection Regulation (GDPR). Further, the development process of the Corona Health platform followed risk-based software validation according to IEC 62304 and IEC 82304 standards for medical device software and healthcare applications as well as the GAMP 5 regulations (standard work of the pharmaceutical industry)^16^.

Participants were recruited through self-selection via the publicly available Corona Health mHealth app on Google Play and the Apple App Store, launched on July, 2020. The app was promoted through social media channels (Twitter) and newsletters. Participants provided informed consent digitally through the onboarding process of Corona Health before participation, which included detailed information about data collection procedures, anonymization protocols, and the voluntary nature of participation. All participants were required to consent to data collection before proceeding to study enrollment.

The privacy policy explicitly informed participants that anonymized findings may be published in scientific journals for research purposes. Importantly, the study employed an anonymous-by-design approach: no personally identifiable information (e.g., names, addresses, email, phone numbers) was collected or stored, and GPS coordinates were limited to 11.1 km resolution. Prior to public data deposition, additional anonymization measures were undertaken in consultation with the institutional data protection officer, including removal of potentially identifying variables and masking of low-frequency demographic categories. The released dataset constitutes anonymized data that cannot reasonably be linked to identifiable individuals.

## Data Records

The dataset comprising the baseline (Baseline.csv) and repeated EMA responses (EMA.csv) can be found at **B2SHARE (EUDAT)**^8^.

The repository also includes sensor data (GPS/APP_Baseline/EMA.csv; i.e., GPS and app usage statistics) collected during the responses and a detailed description of all components from the baseline and repeated EMA that can be found in a comprehensive codebook (Codebook_Baseline_EMA.xlsx).

### Codebook for Baseline and EMA Questionnaires

The codebook provides the structural template for both the baseline and repeated EMA questionnaires used within Corona Health. It is available as a multi-sheet Excel file (Codebook_Baseline_EMA.xlsx), with one sheet dedicated to each version of the questionnaire. Each row in the codebook represents an individual element (e.g., question, headline, or page break), and each column defines specific metadata associated with that element.

The columns are structured as follows:

- **Column A** - elementtype: Defines the type of element in the questionnaire (e.g., pagebreak, headline, text, or question).
- **Column B** - questiontype: Specifies the response format for questions (e.g., SingleChoice, MultipleChoice, Scale, Knob).
- **Columns C-E** - min, max, step: Numeric configuration parameters used primarily for scaled or slider-based inputs (e.g., setting the response range and increment).
- **Column F** - required: Indicates whether a response is mandatory (true or false).
- **Column G** - label: Unique variable name used for storing and linking participant responses in the baseline and EMA files.
- **Column h** - item_de: German-language version of the question text displayed in the app.
- **Columns I-O** - answer_1 to answer_7: Encoded answer options for multiple-choice and single-choice formats, including both numeric codes and display labels.

The Excel file maintains this structure consistently across multiple languages. The first columns until column O reflect the German version (item_de), while subsequent columns provide translations for all supported languages (e.g., English, Spanish, French, Hungarian, Italian, Russian, Serbia). The same structure is applied to the codebook sheet representing EMA questionnaires.

**Fig. 1:**
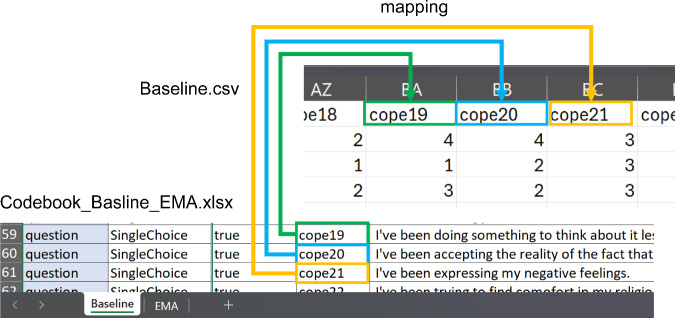
Label Field as Semantic Identifier in Data Files.

The label field (Column G) provides the semantic identifier used to link response values across all datasets (e.g., cope1, phq9_1, whoqol_env1; see Fig. 1). This naming system allows correct mapping between questions in the codebook and their corresponding values in the baseline and EMA responses.

### Structure of the Baseline and repeated EMA Questionnaire

**Fig. 2:**
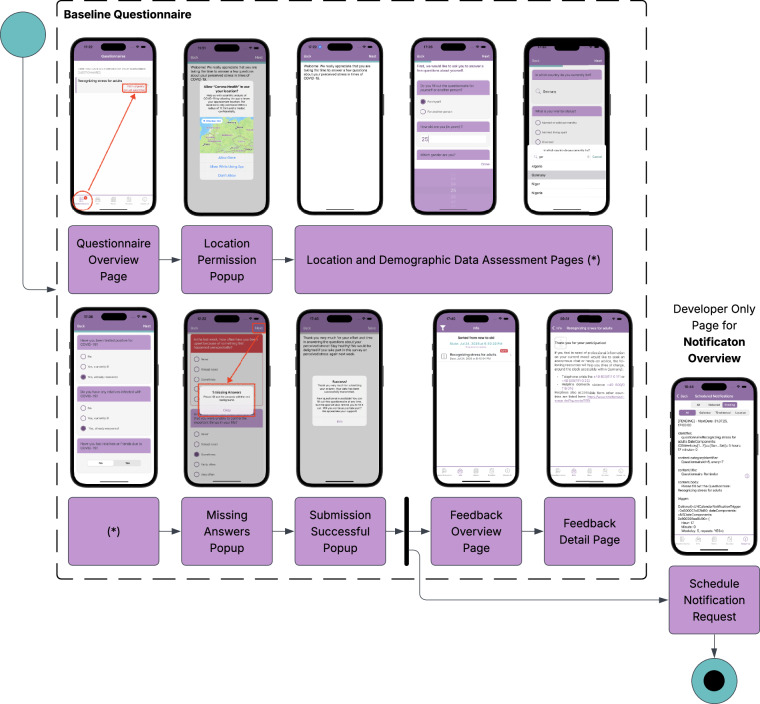
Flowchart of the Baseline Questionnaire.

The baseline questionnaire is structured into 12 consecutive pages, each defined by a pagebreak element in Column A (elementtype) of the codebook. These pagebreaks reflect the logical flow and user experience of Corona Health during questionnaire administration. Figure 2 illustrates the flowchart an user experienced during the answering of the initial baseline questionnaire (see^7^ for more technical information).

Each page corresponds to a specific thematic block, covering core constructs from validated instruments as well as custom items relevant to the COVID-19 context. Note that the following page descriptions highlight the primary instruments and constructs, additional items within each thematic block are fully documented in the codebook. In general, the grouping of items into pages was based on thematic and conceptual similarity rather than empirical factor structures, with the primary goal of creating a coherent user experience and logical flow for participants. For example, Page 7 combines the SOEP loneliness items with the PHQ-9 depression scale, as both assess aspects of psychological and social well-being. This page-level organization was designed for questionnaire administration purposes and does not imply a unified underlying construct. The pages are defined and structured as follows:

- **Page 1 - Study Introduction**: Introduction and welcome message, providing general study information and instructions.
- **Page 2 - Background and Screening**: Includes initial screening (e.g., whether the questionnaire is completed for oneself or another person), followed by questions on demographic background, household characteristics, socioeconomic status, and direct COVID-19-related impacts.
- **Page 3 - Health and Well-being**: Covers general health status using the Mini European Health Module (MEHM)^33^, family well-being, experiences of interpersonal violence (adapted from PHQ-D)^34,35^, and perceived stigma using items from the Inventory of Stigmatizing Experiences (ISE)^36^.
- **Page 4 - Coping Strategies**: Contains the full 28-item *Brief-COPE Inventory* (Coping Orientation to Problems Experienced)^37^, assessing individual coping strategies in response to stress.
- **Page 5 - Personality Traits**: Administers the 10-item version of the *Big Five Inventory* (BFI-10)^38^, which measures personality traits across five domains.
- **Page 6 - Pandemic-related Stressors**: Includes additional PHQ-D items focused on psychosocial stressors relevant to the pandemic context (e.g., concerns about income, health, or social connection)^34,35^.
- **Page 7 - Emotional Well-being**: Assesses loneliness using items from the German Socio-Economic Panel (SOEP)^39^ and depressive symptomatology with the PHQ-9 scale^40^.
- **Page 8 - Panic and Anxiety Symptoms**: Screens for panic disorder symptoms using the PHQ-PD module^41^ and evaluates anxiety levels with the Generalized Anxiety Disorder 7-item scale (GAD-7)^42^.
- **Page 9 - Quality of Life**: Administers the WHOQOL-BREF instrument (World Health Organization Quality of Life), covering physical, psychological, social, and environmental well-being^43^.
- **Page 10 - Sleep and Lifestyle Factors**: Includes the ISI-7 (Insomnia Severity Index) to assess sleep quality and difficulties^44^, and items about physical activity levels and alcohol use.
- **Page 11 - Support Needs and Resilience**: Captures participants’ unmet support needs and documents meaningful or positive experiences during the pandemic.
- **Page 12 - Closing and Feedback**: Presents a closing thank-you message and allows participants to leave optional free-text feedback.

Each page was presented sequentially in Corona Health, and the grouping into thematic blocks facilitates later analysis by construct. All questions are labeled in Column G of the codebook (label), which provides the corresponding variable names for linking responses in the dataset.

**Fig. 3:**
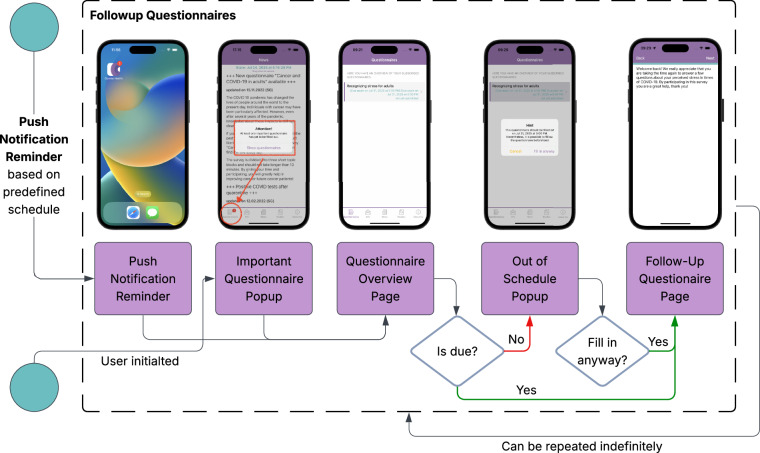
Flowchart of the Repeated EMA Questionnaire.

The repeated EMA questionnaire consists of 10 pages, each marked also by a pagebreak entry in Column A (elementtype) of the EMA sheet in the codebook file. While thematically similar to the baseline, the EMA version includes a reduced number of items, focusing on recurring and longitudinally relevant domains. The selection of EMA items was guided by two primary criteria: (1) minimizing participant burden by reducing questionnaire completion time to approximately 10 minutes, and (2) prioritizing constructs expected to show meaningful within-person variability over time (e.g., depressive symptoms, anxiety, quality of life) while omitting stable trait-like measures assessed only at baseline (e.g., personality traits via the BFI-10, coping styles via the Brief-COPE). The flowchart of the repeated EMA questionnaire is depicted in Fig. 3 (see^7^ for more technical information).

The questionnaire is organized as follows:

- **Page 1 - Session Introduction**: Introduction and welcome message displayed to participants at the start of the session.
- **Page 2 - Current Context and Well-being**: Gathers current living situation, recent pandemic-related experiences, and includes questions from the MEHM and on perceived family climate.
- **Page 3 - Pandemic-related Stressors**: Focuses on psychosocial stressors, adapted from the PHQ-D.
- **Page 4 - Emotional Well-being**: Administers the Loneliness Scale items from the SOEP to assess perceived social isolation and includes the PHQ-9 scale, used to measure depressive symptoms and their severity over the past weeks.
- **Page 5 - Panic and Anxiety Symptoms**: Contains the PHQ-PD module for panic symptoms and the GAD-7 scale to assess general anxiety.
- **Page 6 - Quality of Life**: Includes a shortened version of the WHOQOL-BREF instrument (i.e., EUROHIS-QOL^45^ to measure quality of life across key domains.
- **Page 7 - Sleep and Lifestyle Factors** : Captures responses on the ISI-7 as well as physical activity and alcohol use.
- **Page 8 - Support Needs and Resilience**: Asks participants about support needs and allows them to reflect on positive experiences during the pandemic period.
- **Page 9 - Closing and Feedback**: A thank-you and closing screen, offering participants the opportunity to leave open feedback.

The structure of the EMA questionnaire mirrors that of the baseline, with each item defined in the codebook by its metadata. Specifically, Column G (label) provides the unique variable name used to link responses to corresponding entries in the EMA response file (EMA.csv).

### Structure of Baseline and EMA Responses

The baseline questionnaire responses are stored in the file Baseline.csv. Each row corresponds to a single participant’s completed baseline assessment. The columns are structured as follows:

- **Column A** - user_id: Anonymous identifier assigned to the participant upon enrollment.
- **Column B** - collected_at: Timestamp indicating when the baseline questionnaire was submitted (in ISO 8601 format).
- **Columns C-EU** - Questionnaire responses: Each of these columns corresponds to a specific item in the baseline questionnaire. The variable names used in the column headers are defined in Column G (label) of the codebook and reflect the semantic content of the questions (e.g., phq9_1, cope5, whoqol_env2; see Fig. 1).
- **Column EV** - client_os: Operating system of the participant’s mobile device (e.g., android, ios).
- **Column EW** - client_device: Device model or type (e.g., Google Pixel 5, iPhone 12).

**Fig. 4:**
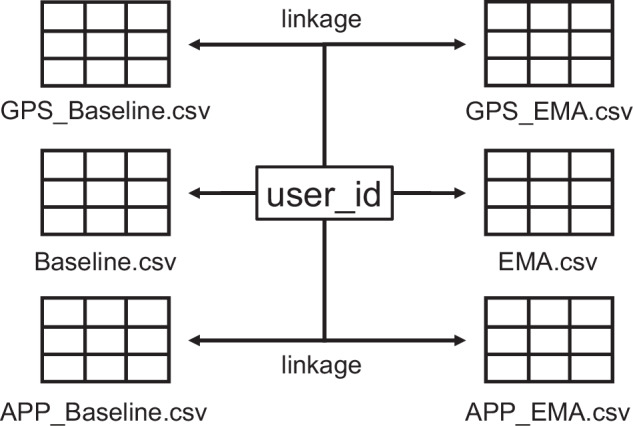
user_id as Linkage Between Data Files.

The user_id is consistently used across other data components (e.g., GPS and app usage) to enable linkage while maintaining privacy (see Fig. 4). Each response column (C-EU) is mapped directly to the respective questionnaire item as described in the codebook. This enables correct identification of question wording, response format, and associated scale parameters. The response structure supports multilingual alignment, as the underlying variable labels are language-independent and consistent across translations.

The repeated EMA responses are stored in the file EMA.csv. Each row represents a single follow-up assessment submitted by a participant. The dataset is organized as follows:

- **Column A** - user_id: Anonymized identifier assigned to each participant. This identifier is consistent across datasets and can be used to link EMA responses to baseline data and sensor records.
- **Column B** - collected_at: Timestamp indicating when the EMA questionnaire was submitted (ISO 8601 format).
- **Columns C-BW** - Questionnaire responses: These columns contain participants’ answers to the EMA items. Each column corresponds to a specific questionnaire variable defined in Column G (label) of the codebook. Variable names are consistent across language versions and represent the semantic content of the item (e.g., phq9_1, whoqol_phys1, isi7_3; see Fig. 1).
- **Column BX** - client_os: Operating system of the participant’s device (e.g., android, ios).
- **Column BY** - client_device: Device model or type used by the participant at the time of questionnaire completion (e.g., Samsung Galaxy A52, iPhone 13).

Equal as in baseline, the response variables (Columns C-BW) are directly linked to the codebook entries using the unique labels defined in Column G.

### GPS Location Data (Android and iOS)

Coarse-grained GPS location data were collected from participants who granted explicit location permission at the time of questionnaire completion. These data were recorded once per submission and are stored in a separate file (GPS_Baseline/EMA.csv). The following columns are included:

- **Column A** - user_id: Anonymous participant identifier that can be used to match GPS records to corresponding questionnaire entries.
- **Column B** - sensordata_collected_at: Timestamp indicating when the GPS data were recorded, typically matching the time of EMA submission.
- **Column C** - sensordata_altitude: Altitude in meters above sea level as reported by the device’s GPS sensor (if available).
- **Column D** - sensordata_longitude: Longitude in decimal degrees, rounded to 0.1^°^ to ensure spatial privacy (approx. 11.1 km resolution).
- **Column E** - sensordata_latitude: Latitude in decimal degrees, similarly rounded to 0.1^°^.

No continuous tracking was performed; GPS coordinates were obtained only at the time of response submission. The spatial resolution was intentionally limited to preserve user privacy and comply with GDPR. These data allow for coarse-grained spatial analyses, such as linking questionnaire responses with regional-level variables (e.g., urban density, local COVID-19 infection rates), while minimizing re-identification risk.

### App Usage Statistics (Android only)

App usage data were collected from Android users who granted explicit sensor permission and are available in respective files (APP_Basline/EMA.csv) as structured columns prefixed with appdata_. These data were recorded once per response submission, corresponding to the day of participation. The columns include general screen usage metrics as well as detailed statistics for the five most frequently used apps on that day.

The following columns are included (column letters refer to the APP_Basline/EMA.csv):

- **Column A** - user_id: Anonymous user identifier (also used for questionnaire data).
- **Column B** - appdata_apps: JSON object listing all apps used on the day of submission, including social media and top 5 apps.
- **Column C** - appdata_beginTime: Timestamp (Unix format) of the first app usage event on the day.
- **Column D** - appdata_collected_at: Timestamp when app usage data were collected (i.e., questionnaire submission time).
- **Column E** - appdata_endTime: Timestamp (Unix format) of the last app usage event on the day.
- **Column F** - appdata_screenTime_activeTimes: JSON or structured list of intervals during which the phone was actively used.
- **Column G** - appdata_screenTime_useTimes: Total screen-on time across all apps.
- **Column H** - appdata_sleepTimes: Inferred inactive periods, approximating rest or sleep phases (JSON or range format).

For each of the five most frequently used apps, the dataset contains the following fields, repeated in blocks of four columns (Columns I-AB):

- **Column I, M, Q, U, Y** - appdata_top5Apps_appX_packageName: Package name of app X (e.g., com.whatsapp).
- **Column J, N, R, V, Z** - appdata_top5Apps_appX_completeFGServiceUseTime: Time app X spent running in foreground service mode.
- **Column K, O, S, W, AA** - appdata_top5Apps_appX_completeUseTime: Total foreground usage time for app X.
- **Column L, P, T, X, AB** - appdata_top5Apps_appX_dailyValues: Encoded JSON or structured summary of hourly usage or usage events for app X.

App names are reported as Android package identifiers. All app usage data are aggregated per day and linked to each questionnaire entry. These columns follow the user metadata and precede the questionnaire responses in the dataset. In general, they are only populated if app usage permission was granted by the participant.

## Technical Validation

**Fig. 5:**
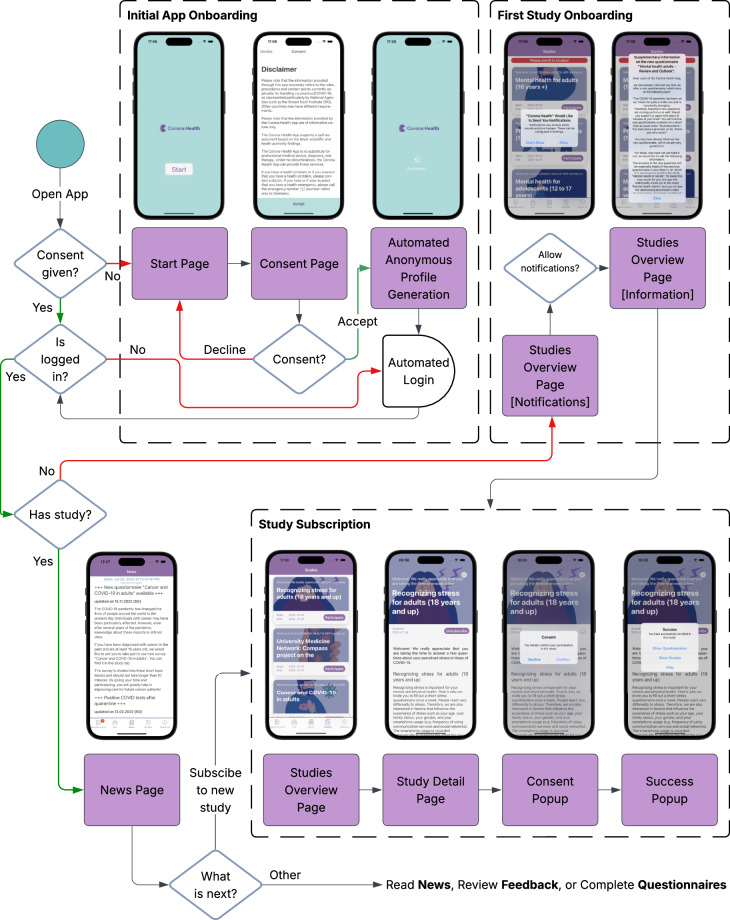
Flowchart of the Onboarding Process.

The Corona Health mHealth app incorporated multiple layers of technical validation to ensure the accuracy, consistency, and reliability of the collected data. Upon initial use, participants were required to authenticate themselves through a registration process, during which the app automatically generated and managed an anonymized authentication account. Users did not need to provide any credentials. This process generated a unique anonymous identifier that was used to enforce data integrity rules across the platform. Figure 5 shows the onboarding process for an user when starting the app and registering for a study (see^7^ for more technical information).

Technically, the system was designed to guarantee that certain data could only be submitted once per participant. In particular, the baseline demographic data and initial mental health assessments were locked to a single submission per user, while repeated EMA responses were versioned and linked to the same identifier, enabling longitudinal tracking without redundancy.

All server-side endpoints that perform write operations (e.g., submitting questionnaire responses or sensor data) enforced a multi-layered validation pipeline before any data were persisted. First, the system verified authentication, confirming that the incoming request originated from a registered user with a valid anonymous session token. Second, an authorization check ensured that the authenticated user held the appropriate privileges to perform the requested operation (e.g., submitting a baseline response only if no prior baseline existed for that user, or submitting an EMA response only if the user was enrolled in the corresponding study). Third, a syntactical validation step confirmed that the JSON payload transmitted to the server conformed to the expected structural format, rejecting malformed or incomplete request bodies before further processing.

Beyond structural integrity, two layers of semantical validation were applied: the system first verified that all fields marked as required in the questionnaire schema were present and non-empty (i.e., that all mandatory items had been answered), and then checked whether the submitted values fell within the permissible ranges defined for each question type. For instance, Likert scale items were validated against their predefined minimum and maximum values, slider-based inputs were checked against their configured range and step parameters, and single-choice items were verified against the set of allowed response codes as specified in the codebook. Responses failing any of these validation steps were rejected by the API and not stored in the database, ensuring that only structurally and semantically valid data entered the system.

This robust validation framework was implemented within a secure RESTful API architecture, based on the TrackYourHealth platform^15^, which supports version-controlled survey deployment, multilingual item consistency, and real-time rule enforcement. Together with compliance to GDPR, MDR, and IEC 62304/82304 software validation standards, these technical safeguards ensured the methodological rigor and quality of the data collected throughout the multi-year study period.

In addition to the real-time validation mechanisms enforced during data collection, several post-export quality assurance steps were performed on the released dataset. It wasverified that all column headers in Baseline.csv and EMA.csv match the corresponding label entries in the codebook, ensuring correct harmonization between questionnaire definitions and stored responses. Cross-file linkage integrity was confirmed by checking that all user_id values in the sensor data files (GPS_Baseline/EMA.csv, APP_Baseline/EMA.csv) have corresponding entries in the questionnaire response files. Missing data patterns were examined across all response variables; item-level missingness is present due to participant attrition, skipped non-mandatory items, and varying engagement over time. The temporal distribution of responses was inspected to confirm that all timestamps fall within the documented collection period (i.e., July 2020 to January 2025) and that no implausible patterns such as duplicate timestamps or pre-launch submissions are present.

The reliability and scientific utility of the dataset are further demonstrated by its prior use in peer-reviewed research. A total of nine publications have already drawn on the dataset described in this data descriptor (see Table 1), demonstrating its value across a range of psychological, behavioral, and digital health research domains.

## Usage Notes

Several aspects should be considered by researchers aiming to reuse the dataset effectively. The data are provided in structured files, including baseline and repeated EMA responses (Baseline/EMA.csv), accompanied by a comprehensive multilingual codebook (Codebook_Baseline_EMA.xlsx). The codebook defines each item, question format, and predefined answer options across all supported languages, facilitating consistent variable mapping.

In general, Tables S.1 and S.2 in the Supplementary Materials provide an overview of participant demographics and study engagement. The baseline sample (n = 2,704) was predominantly female (54.3%), German nationals (98.0%), and in a partnership (60.6%). The age distribution was concentrated in the 25–54 age range (66.8 %), with smaller representation among younger adults aged 18–24 (13.0 %) and older adults aged 65 and above (4.5 %). The majority of participants held academic-level qualifications (42.1 %), and most reported no COVID-19 infection at baseline (92.6 %). Regarding platform use, Android devices were more common (66.9 %) than iOS (33.1 %), and 1,981 participants granted GPS tracking permission while 416 Android users permitted app usage tracking. Of the 1,488 participants who completed at least one EMA, engagement varied considerably, with a median of 8 responses per participant (range: 2–196). Response frequency was highest during 2020–2021 (12,254 responses combined), declining substantially in subsequent years as the pandemic progressed.

Researchers should account for this temporal distribution and the potential for selection bias when interpreting findings or generalizing results. Compared to the general German adult population, women are slightly overrepresented, while older adults aged 65 and above are substantially underrepresented. The sample is considerably more educated and almost exclusively comprises German nationals^46^. These characteristics are consistent with biases commonly observed in voluntary smartphone-based research, where younger, more educated, and more digitally engaged participants tend to be overrepresented, while older adults and those with migration backgrounds are underrepresented^47^. Researchers should exercise caution when generalizing findings to the broader German population, particularly to older adults, individuals with lower educational attainment, and those with migration backgrounds.

Due to the longitudinal design, EMA responses were completed at varying intervals. Each response includes a timestamp, but time gaps between assessments differ across participants and should be accounted for in temporal analyses. Additionally, missing data are present due to participant attrition or skipped items. Researchers are encouraged to examine missingness patterns and apply suitable imputation or exclusion strategies. In rare cases, values may be present in the dataset that fall outside the allowed response ranges as defined in the codebook (e.g., invalid scale values or out-of-range categorical codes). These anomalies likely reflect app-level data entry errors or unexpected client-side behavior and should be identified and handled during data preprocessing.

Sensor data are available for participants who explicitly granted permission at the time of questionnaire completion. These data include coarse-grained GPS coordinates corresponding to an approximate spatial resolution of 11.1 km and app usage statistics, the latter collected only on Android devices. Both types of sensor data were captured opportunistically and linked to individual questionnaire submissions rather than through continuous tracking. At baseline, 1,981 participants granted GPS tracking permission, while 416 Android users permitted app usage tracking. App usage data include total daily screen time, foreground usage durations for the five most frequently used and predefined social media apps, foreground service (background) activity, and intervals of activity and inactivity that can approximate behavioral routines such as sleep. These mobile sensing features provide valuable context for interpreting mental health assessments and facilitate analyses of digital behavior patterns in relation to psychological states. However, researchers should note that app usage statistics are available exclusively for Android users, limiting generalizability of findings derived from these data to approximately two-thirds of the sample. Analyses combining app usage with mental health outcomes should account for this platform-specific availability and consider potential selection effects associated with Android versus iOS users.

Importantly, researchers should note that GPS coordinates were collected only at the time of questionnaire submission, not through continuous tracking. As such, the location data reflect where participants completed assessments rather than comprehensive mobility behavior. This design is well-suited for linking mental health responses to regional-level contextual factors (e.g., local COVID-19 incidence rates, urban versus rural environment, regional socioeconomic indicators) but is not appropriate for analyses requiring true mobility patterns, movement trajectories, or home range estimations. Studies investigating associations between GPS-derived mobility indices and mental states would require continuous location sampling, which was not implemented in Corona Health due to privacy considerations and battery consumption constraints. Suitable analyses with the available GPS data include examining whether regional characteristics predict mental health outcomes, comparing urban versus rural respondents, or linking responses to publicly available area-level data (e.g.,^21^). In contrast, researchers should avoid inferring individual mobility behavior, social contact patterns based on location changes, or time spent at home versus away from the single-timepoint GPS measurements.

To ensure participant anonymity and adhere to GDPR regulations, certain responses were removed or generalized prior to data release. For example, items such as profession in healthcare and low-frequency demographic characteristics were excluded or masked to prevent potential re-identification. These modifications are described in the Data Records section and should be taken into account when conducting subgroup analyses.

No specific scripts or software are required to analyze the dataset. However, it is recommended to use data manipulation tools such as Python (e.g., pandas) or R (e.g., tidyverse) for efficiently handling the structured data, particularly when aligning participant responses with the corresponding questionnaire items defined in the codebook.

For technical details regarding the architecture, implementation, and methodological framework of Corona Health, researchers are referred to related publication^7^. In addition, it is recommended to make an overview of related studies that have utilized this dataset, as they may offer methodological insights and comparative benchmarks for secondary analyses (see Table 1).

Note that some responses from the original responses were removed or modified due to the risk of participant re-identification when considering combinations of certain items. For example, the baseline questionnaire included a question about whether the respondent worked in a healthcare profession. Due to the specificity of this question and the relatively small number of individuals it applies to within the sample, it posed a potential risk of re-identification, especially when considered alongside other sociodemographic variables such as age, gender, and geographic region. To mitigate this risk and comply with data protection regulations, responses to this item were removed in the shared dataset. Additionally, 22 participants reported having a diverse gender identity. Given the low frequency of this response and its potential to allow indirect identification, these participants were masked, and their gender category was randomly replaced with either male or female in the publicly available dataset. This modification was performed solely for data protection purposes and does not reflect the original responses of the affected individuals. This approach follows a practice used by the Federal Statistical Office of Germany, where cases with the gender categories “diverse” or “unknown” are redistributed to the categories “male” and “female” using a predefined recoding procedure, as these categories currently cannot be reported separately for methodological reasons^48^. Furthermore, the data were not deleted, as doing so would raise ethical concerns: collecting information from gender-diverse individuals only to later exclude it from analysis would be inappropriate and contrary to the principles of inclusive and respectful research practice.

## Supplementary information

Supplementary Information

## Data Availability

The complete dataset can be accessed freely and publicly at B2SHARE (EUDAT): https://b2share.eudat.eu/records/cgf63-kme28.

## Code availability

No custom code was used in the generation, processing, or analysis of the dataset described in this manuscript. The dataset represents a raw data export from the Corona Health platform. Apart from the anonymization steps described, such as the removal or generalization of potentially identifying variables, no further processing or transformation was applied.
