# Supplementary material for: Longitudinal mental health data collected via the Corona Health smartphone app during COVID-19
Source: Sci Data. 2026 Mar 11;13:392. doi: 10.1038/s41597-026-07015-7 (PMC12992553; doi:10.1038/s41597-026-07015-7)
Supplement: Supplementary file 1 — Supplementary Information [file 41597_2026_7015_MOESM1_ESM.pdf]

Table S.1. Basic Demographic Information from the Baseline Questionnaire

Category	Sub-category	Count (n)	Percentage (%)
Age Group	18 - 24	352	13.0
	25 - 34	623	23.0
	35 - 44	665	24.6
	45 - 54	520	19.2
	55 - 64	422	15.6
	65 - 74	104	3.9
	75 +	17	0.6
Gender	Female	1,469	54.3
	Male	1,235	45.7
Nationality	German	2,649	98.0
	Other	55	2.0
Family Status	Partnership	1,639	60.6
	Single	1,065	39.4
Education/ Profession	No Degree	412	15.2
	School Education	887	32.8
	Higher Level	266	9.8
	Academic Level	1,139	42.1
COVID-19 Status	No Infection	2,505	92.6
	Recovered	125	4.6
	Currently Infected	74	2.7

Table S.2. Study Engagement & Temporal Data

Category	Metric	Count (n)
Total Users	Baseline	2,704
	EMA	1,488
Mobile OS	Android	1,811
	iOS	893
Tracking Permissions	GPS	1,981
	App (Android)	416
EMA Engagement	Minimum Responses	2
	Maximum Responses	196
	Median Responses	8
Response Frequency	2020	5,889
	2021	6,365
	2022	1,679
	2023	241
	2024	66
	2025	5
